# Sound effects have only minor contribution to perceptions of anthropomorphism and animacy of simple animated shapes

**DOI:** 10.1177/20416695251315382

**Published:** 2025-02-02

**Authors:** KC Collins, Maya Murad, Adel Manji

**Affiliations:** 6339 Carleton University, Canada

**Keywords:** animacy, anthropomorphism, sound, sound effects, shapes, geometric, animation

## Abstract

While studies of anthropomorphism have spanned many decades, there is little evidence of the role that sound effects may play. We present two studies into sound's influence on perceptions of anthropomorphism and animacy using simple geometric animated shapes. For the first study, conducted on 149 participants, we simplified the animation to just two “bumping” squares. Study Two recreated the Heider-Simmel study of 1944, and was conducted on 250 participants under five conditions: without sound, and with one of two different sound types (interface sounds and “anthropomorphic” robot sounds) with two stereo modes (fixed in stereo position, or binaurally panned with the movement). We had participants answer both the Individual Differences in Anthropomorphism Questionnaire and the Godspeed Questionnaire, with three additional questions added. Results showed that the sound had a minor impact on anthropomorphism and potency in Study One, but did not impact animacy. Study Two showed no significant effect on anthropomorphism or animacy, but did show an impact on perceived intelligence and perceptions of activity.

## How to cite this article

Collins, K.C., Murad, M., & Manji, A. (2025). Sound effects have only minor contribution to perceptions of anthropomorphism and animacy of simple animated shapes. *i–Perception*, 16(1), 1–25. https://doi.org/10.1177/20416695251315382

Anthropomorphism is the tendency towards ascribing non-human entities to human behaviors, intentions, feelings, or perceptions. Interest in anthropomorphism has grown rapidly with the rise of attempts at creating natural human-computer interfaces, AI-enabled voice assistants, and consumer-facing robots. Anthropomorphism has been of particular interest when it comes to voice assistants (e.g., Beirl et al., 2019; Lee et al., 2020; Motalebi et al., 2019; Pradhan et al., 2019; Voit et al., 2020; Wagner & Schramm-Klein, 2019). There are many advantages to anthropomorphizing a product for example: it increases trust (de Visser et al., 2016), likeability (Walters et al., 2008), acceptance, and attachment (Eyssel et al., 2012, 2012).

**Figure 1.**
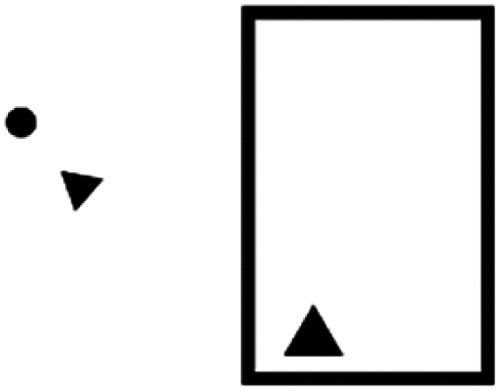
Replication of the Heider and Simmel (1944) animation.

Numerous studies have highlighted that our inclination to anthropomorphize is influenced by multiple factors, including our gender, age, personality, socio-cultural background, and social situation, particularly loneliness (Eyssel & Reich, 2013). For instance, research by Chandler and Schwarz (2010) suggests that the longer we interact with an object, the more likely we are to anthropomorphize it and develop an attachment. Epley et al. (2007) offer a three-factor theory to explain anthropomorphism: the accessibility and applicability of “anthropocentric knowledge” (a form of reasoning where people use their own human-centered knowledge to predict animal behavior); the motivation to comprehend behavior (effectance motivation); and the desire for social contact. Morrison and Ziemke (2005) assert that humans can easily anthropomorphize even simple animated agents, making personality trait attributions to geometric shapes or point-light figures with minimal provocation. They note that it takes little to interpret a triangle as “chasing” a square, or to perceive the triangle as “mean” and the square as “frightened” (see also Tremoulet and Feldman, 2000). This effect was shown decades earlier in a famous study by Heider and Simmel (1944), which used a short animation of moving shapes. The animation is approximately two minutes long and consists of three figures (a large triangle, a small triangle, and a small circle) that move around a rectangular shape (a “room” with a “door” that opens and closes; Figure 1). In the original study, participants were asked to describe and interpret the actions and interactions of the shapes. Participants consistently interpreted the movements in anthropomorphic terms. For example, a triangle might be perceived as chasing or bullying a circle, or two triangles might be seen as interacting playfully. There have been multiple critiques of the verbalization requirements of the original study (see Rasmussen and Jiang, 2019).

A related concept that is very close to anthropomorphism is animacy, which refers to “lifelikeness,” or the extent to which an entity or object is perceived as alive or having lifelike qualities (Bartneck et al., 2009). Unlike anthropomorphism, which involves attributing *human*-like emotions, intentions, or behaviors to non-human objects, animacy focuses on perceived alive-ness. Thus there is considerable overlap between these concepts, however, the distinction is the degree to which the behavior or intent is perceived as human, rather than merely animate. Movement plays a crucial role in shaping perceptions of animacy (Parovel, 2023). Investigating perceived animacy sheds light on underlying cognitive processes, including pattern recognition, social cognition, and social structures, as well as how people attribute agency and intentionality (Scholl and Tremoulet, 2000). Animacy can also be useful for designers to understand, since understanding animacy perception helps designers create more engaging and intuitive interfaces, by making interactions more natural and relatable. Products that are perceived as having a personality can drive consumer loyalty and engagement (Milman et al., 2020).

Research has largely focused on anthropomorphic tendencies rather than animacy, partly due to the challenges in measuring animacy (Scholl & Tremoulet, 2000), although there have been more recent scales to attempt to measure the phenomenon (see section “Questionnaires”). Tremoulet and Feldman (2000) showed that a single moving object could evoke a sense of animacy based on its movement pattern. Similarly, Gao et al. (2009) in a series of simple animations found that simple shapes that appear to chase each other (such as those in the Heider-Simmel study) are perceived as highly animate.

Research on animacy and anthropomorphism has been primarily focused on visual elements and offers us insights into visual perception. Parovel's recent review (2023) examines the importance of speed, directionality, and interaction of objects in our perceptions of animacy, for example. While anthropomorphism and animacy are clearly associated with visual cues, it is less clear if sound also plays some role in this phenomenon. Sound has been shown to influence other aspects of visual perception, for instance in perceptions of smoothness of animations (Mastoropoulou et al. 2005), and thus it would be useful to understand the influence of sound on anthropomorphism and animacy cues.

In animation and film, even without using human language, animated characters are often given voices that mimic human speech patterns and emotions, making them more relatable and emotionally engaging, and thus potentially more animate and anthropomorphic. Shin and Mattila (2021) conducted a study on prosocial initiatives involving cuteness, examining sound and no-sound conditions using images of human babies paired with sounds of baby laughter. Their findings indicated that sound enhanced prosocial responses towards the babies. However, they did not investigate the effects of sound on inanimate objects or non-human sounds, nor look at the influence on animacy or anthropomorphism.

A paper by Millicent Cooley (1998) based on her master's thesis explored some basic interactions between shapes and sound in a small qualitative study, although did not test for anthropomorphism or animacy explicitly, and only tested on eight participants. She added different types of sound to a series of simple shape animations and asked users how sound influenced their perception of various physical characteristics like weight, density, hollowness, and perceived distance. She noted that since shapes do not have sounds in the everyday world, people were more willing to suspend disbelief when it came to the abstract sounds she associated with these shapes, in comparison to more real-world identifiable staple gun and toilet plunger sounds. In one of her studies, she tested two simple “bumping squares,” where an animation of two squares moving towards each other with a slight bounce animation. Participants were asked “if the various sounds make the squares seem light or heavy, dense or hollow, rough or smooth, large and far away or small and near,” but the study was only qualitative, and only vaguely described (that is, not all results were presented, and it is unclear why specific responses were selected, or if they were representative of all respondents). There are also no details about the sounds used or how they were created, nor is any data provided. We therefore adapted her “bumping squares” animation as the visual basis for Study One, but used our own sounds, with the goal of providing statistical data and specifically testing anthropomorphism and animacy.

Collins and Dockwray (2018) conducted a small pilot study exploring the anthropomorphic tendency of responses to sound and no-sound conditions using a virtual Tamagotchi-style game. A total of 17 players played the game without sound, and 24 with sound, which consisted of simple low-fidelity beeping, similar to the original device. Players spent longer voluntarily playing with sound, however, the results are largely inconclusive, although when asked directly about the sound, the participants said it was more lifelike. Their study relied on qualitative responses. The authors suggest that prosodic use of sound—that is, where the sound effects mimic a kind of verbal quality in terms of rhythm and prosody—would probably increase the effects they found.

Marshall and Cohen (1988) recreated the Heider and Simmel study and added musical soundtracks in two related studies. Participants were tested under one of five conditions: no soundtrack, only one of two different music soundtracks (no visual), or one of two music soundtracks accompanying the film. They used qualitative results in combination with a 16- (shrunk to 13 for the second study) question scale grouped into Evaluative, Potency, and Activity dimensions, developed by Osgood et al. (1957). Evaluative refers to the goodness or badness, or how favourable and pleasant something is (nice/awful, good/bad, beautiful/ugly, pleasant/unpleasant). Potency measured strength or weakness, as relates to power or intensity (weak/strong, powerless/powerful, submissive/aggressive, small/large). Activity referred to perceptions of energy and dynamism (calm/agitated, submissive/aggressive, quiet/restless, fast/slow).

They found that the music altered all of the ratings on the dimensions tested, suggesting that associations created by the music provided context for the interpretation of the action in the film clip. In the first study, run on 25 people, when no music was present, participants described the film characters in a stereotypical way, similar to the original interpretations from Heider and Simmel (1944). For example, the large triangle was often seen as aggressive. However, when music was added (either adagio or allegro), the interpretations and ratings of the characters shifted. The adagio music (slower paced) led to lower agitation ratings for the large triangle and small circle, while the allegro music made the small triangle seem more agitated than in the no-music condition. In the second study, run on 30 participants, the influence of the soundtracks (“weak” and “strong” music) was even more pronounced. With music, the film overall received higher potency and activity ratings compared to the no-music condition, especially with strong music. Music also altered the evaluative dimension, where, for example, weak music made the film seem less strong and active. In contrast, the no-music (film only) condition led to higher evaluative ratings, suggesting that without music, the film was seen more favorably and with a more straightforward interpretation. However, there were 30 participants in the studies, and spreading across the five conditions, this meant that the maximum number of participants in each condition was only six, making any statistical validity questionable.

Kim and Schachner (2021) also investigated the relationship between music and animacy, albeit not with a visual animation. They suggest that music, with its perceived movement and directionality, “activates representations of movement, even when no movement is produced or viewed.” Their study explored whether musical sounds could imply the presence of an agent behind them. While intriguing, it does not directly address how sound contributes to attributing animacy to objects, especially since music typically has a longer duration than sound effects, and has attributes not normally heard in sound effects (repetition/rhythm/tempo, dynamism over time, directionality in melody and harmony, etc.).

Nielsen et al. (2015) studied how the perception of motion in sound effects alone (without a visual correspondent) impacts perceived animacy. They discovered that the sound of a mosquito moving in a binaurally spatialized way along a specific trajectory—demonstrating a behavioral pattern—enhanced the sense of animacy similarly to a moving visual object. Their findings confirmed that the perceived movement of sound alone can influence perceptions of animacy. We therefore added a binaural component to Study Two. It did not make sense to add a binaural element to Study One, since the animation's short visual separation is not enough to render spatialized cues relevant, and the length of the video was too short to make any spatialization appropriate.

In another small study by Komatsu (2005), the researcher explored how participants perceived the attitude of a computer based on its low-fidelity beeps. They discovered that beeps with increasing intonation were perceived as “disagreement,” decreasing intonation as “agreement,” and sounds with a flat melodic contour of longer durations as “hesitation.” This demonstrated that beeps could convey attitudes, thereby anthropomorphizing a simple computer (Komatsu 2005). Nevertheless, this study was limited to what Komatsu described as beeps, but we are not sure of the complexity of the actual sounds as they are not available (were they sine waves, and were the beeps a single frequency, etc.?), and the author did not compare sound to no-sound conditions. Moreover, the study involved a small sample of 20 people, almost exclusively male Japanese participants.

There is also a considerable body of work that explores the impact of a human voice on anthropomorphism of smart devices and robots. Voice has been shown to increase a sense of social presence in interfaces, and users tend to ascribe personalities to objects that have a voice (Evans & Kortum, 2010; Lee & Nass, 2003). The human voice carries elements of age, gender, class, ethnicity, affect, and intent (Schreibelmayr and Mara, 2022). Studies have shown differences in perceptions between human and synthesized voices, with human voices paired with robots achieving a less likeable, uncanny valley effect (Epley, 2018; Schroeder & Epley, 2016).

Luo et al. (2023) used the Godspeed questionnaire (see section “Questionnaires”) to explore anthropomorphism in voice assistants. They found that embodied (e.g., with visual robot body) voices elicited higher anthropomorphic perception. Bakardzhiev (2022) found flat-pitched synthesized voices were less anthropomorphic. Steinhaeusser and Lugrin (2024) likewise tested synthesized and human voices in a robot telling a story, and found anthropomorphism, animacy, and likeability were higher for human voice, but the degree to which they differed depended on the number of different voices in the story told. However, such results are probably not surprising when comparing human and synthetic voices in dialogue-based storytelling with a humanoid figure, and it is unclear if the results would carry over to simple shapes in a non-dialogue animation.

We could find no studies that have measured the impact that sound effects have on perceptions of animacy or anthropomorphism of simple animated shapes. There has been a large amount of work that has been done to recreate the Heider and Simmel study under a variety of conditions, such as with different sized shapes and line weights (Lynch, 1991); in VR (Marañes et al., 2024); in monkeys (Schafroth et al., 2021), with those on the autistic spectrum (Rasmussen and Jiang, 2019); and many more. It is surprising, then, that the use of sound in this animation has never been studied.

In this paper, we adapted aspects of the work described above to investigate the role of sound effects on perceptions of anthropomorphism and animacy of simple shapes. The first study uses Cooley's (1998) bumping squares as a basis for the animation, and the idea to test under different sound conditions. The second recreates the Heider and Simmel animation (1944). For the sounds, we adapted the concept of using some simple effects of real-world sounds from Cooley (1998) for Study One, and for Study Two, synthesized beeps from Komatsu (2005). We would compare those with adaptations of human voice, adapting the idea from Epley (2018). We adapted the idea of exploring binauralization in Study Two from Nielsen et al. (2015). In terms of measures, we adapted two scales known as the Godspeed Questionnaire and the Individual Differences in Anthropomorphism Questionnaire, described below (section “Questionnaires”), and added three questions, or Dimensions, from Marshall and Cohen's (1988) study that were not covered by those questionnaires, in order to compare our results. Using the existing literature as our inspiration, our hypotheses were:

H1(a). Sound effects will contribute to an increase in both animacy and anthropomorphism, as evaluated by an increase in Godspeed scores.

H1(b). Human-based vocal sounds will be a greater contributor to this tendency than non-vocal sounds (real-world, or synthesized sounds).

We then added a second hypothesis for the second study.

H2. Binaurally spatializing sound will increase both animacy and anthropomorphism.

## Study One: Bumping Squares

**Figure 2.**
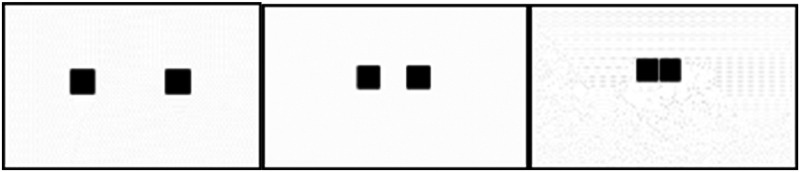
(a, b, c) squares “bump” into each other in simple animation.

Study One used a simple animation of two squares that appear to “bump” into each other, lasting just 5 s long, which was designed based on the description provided by Cooley (1998). The animation was created with a JavaScript animation library called Anime.js (Garnier, 2024; Figure 2 a, b, c), and subsequently converted into videos for the three conditions. The black squares on a white background begin the animation spaced apart and travel inwards to collide with each other, with a slight bounce off each other, before coming to rest.

### Sounds

There were three sound conditions: (1) No sound at all (no-sound), (2) a synthesized robot voice, which we called our “Anthro” sound, approximately resembling a giggle sounding like “hee hee,” and (3) a “simple” sound of a knock, roughly sounding like two blocks of wood with a thin piece of fabric in between them smacking together (simple sound). The Anthro sound was created using a recording of one of the researchers’ voices. The audio was then sped up in Ableton Live, without any warping to preserve its pitch such that the resulting clip was eight semitones higher than it was initially. This audio was then put through a high-pass filter and a phaser effect. The simple sound was created by stacking two recordings created by the research team of knocking on a wooden surface. One recording had a hollower sound and one was more deadened (higher spectral components removed). The sounds’ transients were closely aligned, and some equalization was applied to both. Some compression was also applied as well as a very small amount of convolution reverb. Sounds were synched to the video in post-production and normalized to −3 dB for the video. The sounds can be found with the videos on the Zenodo link (*redacted for peer review).

### Questionnaires

Surveys were constructed in Qualtrics (https://www.qualtrics.com/) and participants were recruited from Prolific (https://www.prolific.com/), a paid research survey service similar to, but of reputably higher quality responses than, Amazon's Mechanical Turk, or MTurk (https://www.mturk.com/ For a comparison of the quality of response between MTurk and Prolific, see Albert and Smilek, 2023). Participants could only participate once. The study contained two attention checks, and those who failed at least one of those checks were removed from the study's data. All other participants were included.

Participants first filled out the Individual Differences in Anthropomorphism Questionnaire scale (IDAQ), which is designed to assess our individual tendencies to anthropomorphize (Waytz et al., 2010). There are known gender and cultural differences in our tendencies towards anthropomorphism, as well as individual differences (Epley et al., 2007; Waytz et al. 2010), therefore it was necessary to first assess our participants’ general predisposition to anthropomorphize. The IDAQ is a validated scale that consists of fifteen questions such as, “To what extent does the environment experience emotions?,” or “To what extent does the average computer have a mind of its own?” Each question has an 11-point Likert scale response where 0 = “Not at All” and 10 = “Very much.” Numbers are then tabulated for half of the questions, and the result is a number out of a total of 150, where higher scores indicate a greater tendency to anthropomorphize.

After completing the IDAQ, participants watched a randomly selected video from one of the three animation sound conditions, then completed the Godspeed Questionnaire (GSQ) asking about their perception of the squares in the animation. The GSQ is one of the most commonly cited questionnaires in human–robot interaction and was designed primarily to assess our anthropomorphic responses to robots. The GSQ was created to explore anthropomorphism and animacy specifically and has been used extensively in the literature (Bartneck et al., 2009). The GSQ is divided into five categories or dimensions of questions: anthropomorphism, animacy, likeability, perceived intelligence, and perceived safety. Each dimension has five or six questions. The questionnaire has been used hundreds of times since publication (Bartneck, 2023; Bartneck et al., 2009). The GSQ is based on a five-point semantic differential, where participants are asked, “Please rate your impression of [the squares] on these scales,” and are required to mark a response in terms of opposing terminology, such as “unconscious – conscious,” “artificial – lifelike,” “unpleasant – pleasant,” or “fake – natural.” Scores are then averaged within each concept group to get a mean score for the participants.

We added three other question blocks, the dimensions drawn from Marshall and Cohen's (1988) study of music's impact on attitudes towards Heider and Simmel's animated geometric figures. In Marshall and Cohen's study, participants rated responses on a series of semantic differential questions grouped into three dimensions: (1) Evaluative Dimension: Nice/awful, Good/bad, Beautiful/ugly, Pleasant/unpleasant, (2) Potency Dimension: Weak/strong, Powerless/powerful, Submissive/aggressive, Small/large, and (3) Activity Dimension: Calm/agitated, Passive/active, Quiet/restless, Fast/slow. Marshall and Cohen found that these factors were all influenced by music. As described above, they found potency and activity, in particular, were impacted, and that on the evaluative dimension, the soundtrack that received higher potency and activity ratings led to a lower rating of the film overall in this category (that is, the inclusion of music resulted in lower evaluative dimensions/likeability). However, the researchers did not test music's influence on the tendency to anthropomorphize, or if music increased the tendency to attribute animacy to the shapes in the film. We have called this combined survey the Godspeed + questionnaire, for the purposes of this paper.

### Participants

Participants were required to speak English and have a minimum age of 18. We successfully recruited 149 participants from Prolific (see section “Questionnaires”), after one was removed for failing attention checks. Participants took approximately 8 min to complete the surveys and were paid £1.50 (GBP). 48 participants were in the No Sound condition, 51 were in the Simple Sound condition, and 50 were in the Anthro Sound condition.

Participants were first asked for basic demographic information. They were also asked about the listening device used for the study in the form of multiple choice. In terms of audio device used, 61 participants were on laptop speakers, 12 on desktop speakers, 11 on external speakers, 14 on mobile device speakers, and 51 on headphones.

The survey asked participants to indicate their age category, based on standard age groupings. There were 46 participants in the 18–24 age group, 73 in the 25–34 age group, 10 in the 35–44 age group, 9 in the 45–54 age group, and 11 in the 55 + age group (since there was only one participant over 65 we included that in the 55 + for our statistical comparisons).

Given that Prolific tends towards younger users (being an Internet service), based on our past experience, we grouped those over 55 together into one category. Past research found that age was a factor in individual tendency to anthropomorphize (Letheren et al. 2016); however, an ANOVA showed no significant differences in responses to the IDAQ between different age groups (*F*(4, 144) = .165, *p* = .956, *η*^2^ = .005). Likewise, an ANOVA showed no significant differences in IDAQ responses between participants with reported hearing deficits (9 said they had some known hearing deficit, and 3 responded “maybe”), and those with no known hearing deficits (*n* = 137) (F(2, 146) = .060, *p* = .942, *η*^2^=.001).

There were differences in mean IDAQ scores between different regions. 59 participants were from Europe, 1 from Australia, 21 from North America, 2 from South America, and 66 from Africa and the Middle East. For the purposes of this test, Australia and South America were combined into one group, “Other,” due to their low participant count. A one-way ANOVA revealed significant differences in IDAQ scores between regions, *F*(3, 145) = 6.843, *p* < .001, *η*^2 ^= .124. Levene's test for homogeneity of variances was significant, *F*(3, 145) = 4.235, *p* = .007, indicating a violation of the assumption of equal variances. Due to this violation, the Games-Howell post hoc test was chosen, as it does not assume homogeneity of variances and is suitable for unequal group sizes. The post hoc test revealed that Africa and the Middle East (*M* = 67.85, SD = 30.335) had significantly higher IDAQ scores than both Europe (*M* = 47.22, SD = 20.952), *t*(116) = 4.461, *p* ≤ .001, and North America (*M* = 52.19, SD = 20.223), *t*(51) = 2.709, *p* = .044. However, no other significant differences were found. We did not explore ethnicity within these regions, and such a break-down may be interesting to undertake in future studies.

There were 85 participants who identified as women, 61 identified as men, and 3 identified as other. ANOVA results showed no significant differences in IDAQ scores between genders for this study (*F*(2,146) = .956, *p* = .387, *η*^2 ^**^= ^**.013), although women (*M* = 59.99, SD = 25.464) had higher IDAQ scores than men (*M* = 53.82, SD = 30.361) and than those who identified as other (*M* = 51.67, SD = 51.67). While we used the IDAQ scores for our analyses, we did not further separate out the results by demographics, as the impact of specific demographics was not a focus of our study.

### Results of Study One

**Figure 3.**
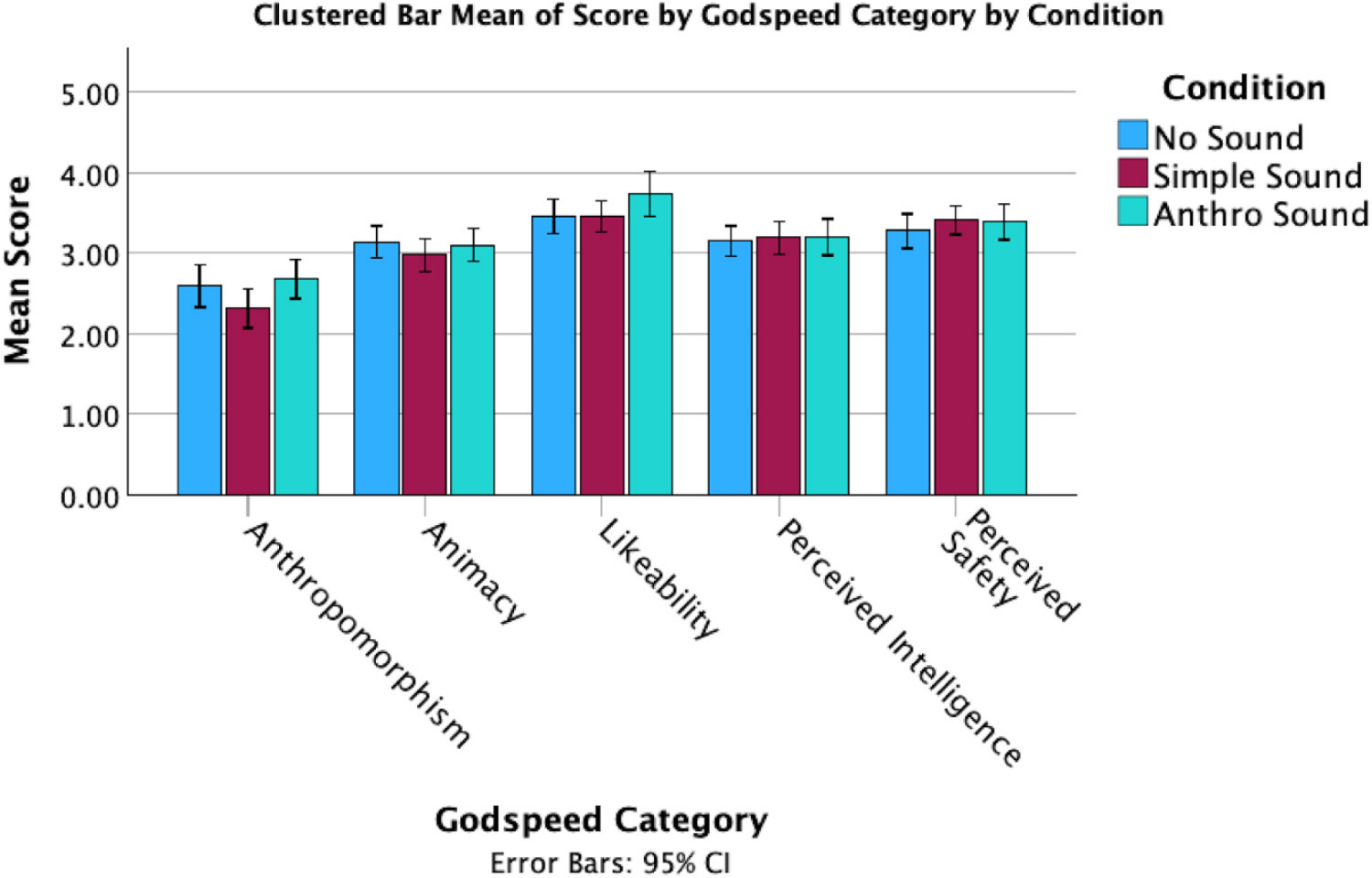
Clustered bar means of godspeed by sound condition.

**Figure 4.**
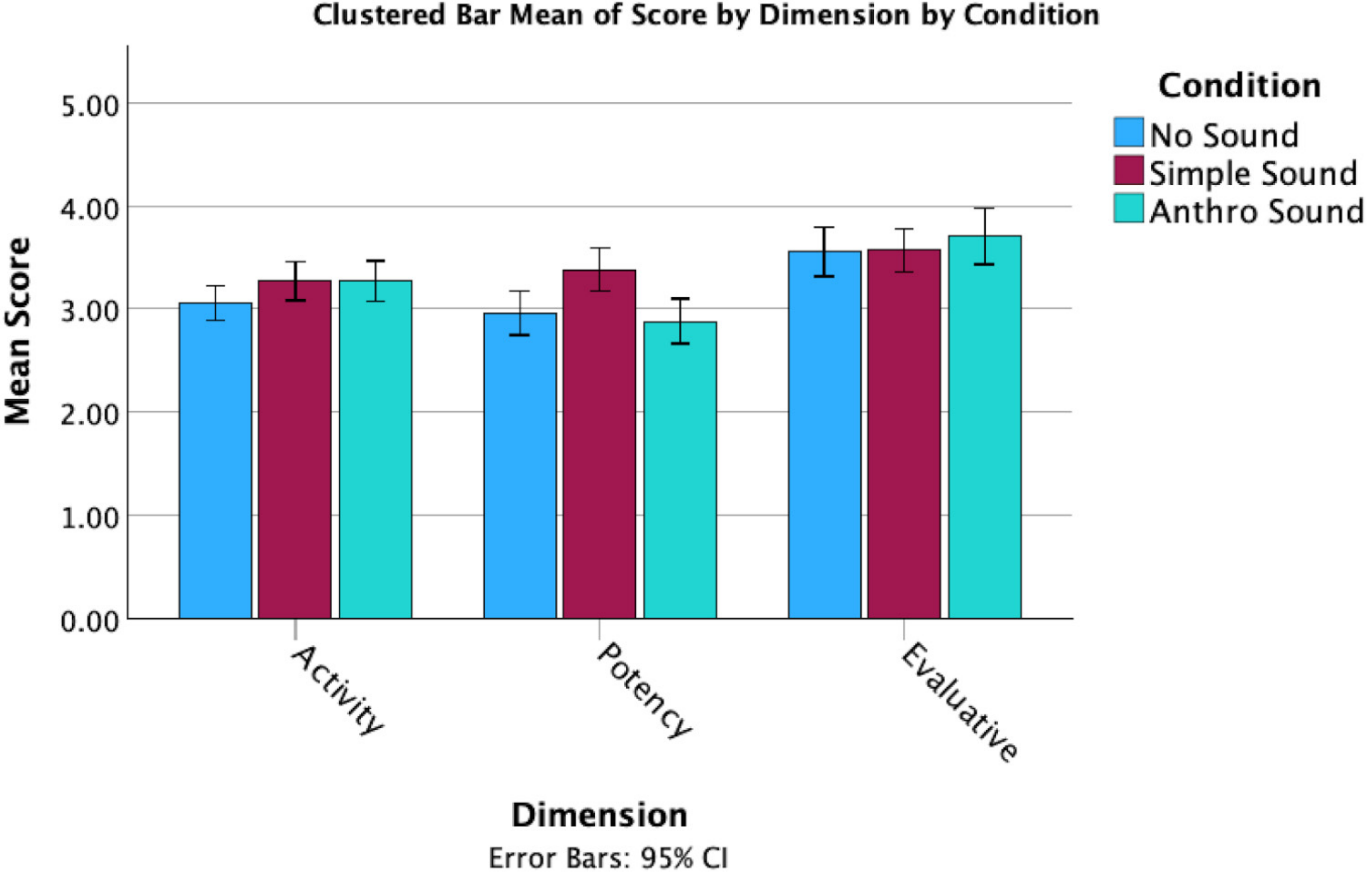
Clustered bar means of dimension scores by sound condition.

In terms of responses on the Godspeed + questions, there were several noticeable differences between the mean scores for the sound conditions (Figures 3 and 4). However, these observations do not take into account the individual differences in tendency to anthropomorphize. While perceived intelligence and safety remained consistent across conditions, the Anthro Sound and No Sound both had higher anthropomorphism, animacy and likeability than the Simple Sound condition.

A statistical analysis was carried out in SPSS Statistics (IBM Corp, 2023). Scatterplots along with Pearson Correlation coefficients indicated that there were significant linear relationships present between the covariate, IDAQ scores, and most of the dependent variables: anthropomorphism (*r*(147) = .49, *p* < .001), animacy (*r*(147) = .419, *p* < .001), likeability (*r*(147) = .194, *p* = .018), perceived intelligence (*r*(147) = .370, *p* < .001), perceived safety (*r*(147) = .292, *p* < .001), evaluative dimension (*r*(147) = .228, *p* = .005), and potency dimension (*r*(147) = .283, *p* < .001), therefore it was appropriate to use an ANCOVA to analyze these variables.

However, through the inspection of scatterplots and a Pearson Correlation coefficient, no linear relationship was found between IDAQ scores and activity dimension scores (*r*(147) = −.004, *p* = .966), suggesting that IDAQ score was not a relevant covariate. Accordingly, we conducted a one-way ANOVA to analyze the effect of condition on activity dimension scores.

For the following ANCOVA tests, the assumption of homogeneity of regression slopes were tested for all variables by examining the interaction between IDAQ scores and conditions, indicating that the slopes were homogeneous: anthropomorphism (*F*(2,143) = .628, *p* = .528), animacy (*F*(2,143) = 1.577, *p* = .210), likeability (*F*(2,143) = .585, *p* = .558), perceived intelligence (*F*(2,143) = .00, *p* = 1.00), perceived safety (*F*(2,143) = 1.011, *p* = .367), evaluative dimension (*F*(2,143) = 1.066, *p* = .347), and potency dimension (*F*(2,143) = .322, *p* = .725).

The normality of residuals was assessed using both Shapiro-Wilk tests and visual inspection of normal Q-Q plots. Most variables showed approximate normal distribution based on the Shapiro-Wilk test: anthropomorphism (*W* = .992, *p* = .559), animacy (*W* = .991, *p* = .425), perceived intelligence (*W* = .988, *p* = .259), perceived safety (*W* = .992, *p* = .617), and potency dimension (*W* = .987, *p* = .187).

However, the Shapiro-Wilk test indicated significant deviations from normality for likeability (*W* = .966, *p* < .001) and the activity dimension (*W* = .980, *p* = .031). Despite this, visual inspections of Q–Q plots suggested that the deviations were minimal and did not display clear patterns of skewness or kurtosis. Normality tests can be sensitive to small deviations, particularly in large samples, which may lead to significant *p*-values even when the overall distribution appears approximately normal (Kozak & Piepho, 2018). Furthermore, ANCOVA is fairly robust to deviations from normality, especially in larger sample sizes (>30) (Olejnik & Algina, 1984; Pallant, 2010). Therefore, based on the Q–Q plot evaluations, an ANCOVA was still performed for the variables with acceptable approximate normality.

Levene's test confirmed homogeneity of variances for most variables: anthropomorphism (*F*(2,146) = 1.528, *p* = .220), animacy (*F*(2,146) = .085, *p* = .919), perceived intelligence (*F*(2,146) = 2.133, *p* = .122), perceived safety (*F*(2,146) = .543, *p* = .582), evaluative dimension (*F*(2,146) = .848, *p* = .430), activity dimension (*F*(2,146) = .927, *p* = .398), and potency dimension (*F*(2,146) = .114, *p* = .893).

For likeability, the assumption of homogeneity of variances was not met, *F*(2,146) = 3.669, *p* = .028, however results from the ANCOVA revealed no statistically significant differences, *F*(2, 145) = 2.630, *p* = .076, partial *η*^2^ = .035.

Additionally, after adjusting for IDAQ scores, results from the ANCOVA revealed no statistically significant differences between conditions for animacy (*F*(2, 145) = .917, *p* = .402, partial *η*^2^ = .012), perceived intelligence (*F*(2, 145) = .640, *p* = .529, partial *η*^2^ = .009), perceived safety (*F*(2, 145) = 1.243, *p* = .291, partial *η*^2^ = .017), or evaluative dimension (*F*(2, 145) = .917, *p* = .402, partial *η*^2^ = .012). Similarly, results from the ANOVA indicated that there were no significant differences in activity dimension scores across the different conditions, *F*(2, 146) = 1.799, *p* = .169, *η*^2^ = .024 (as a reminder, no linear relationship was found between IDAQ scores and the activity dimension).

**Figure 5.**
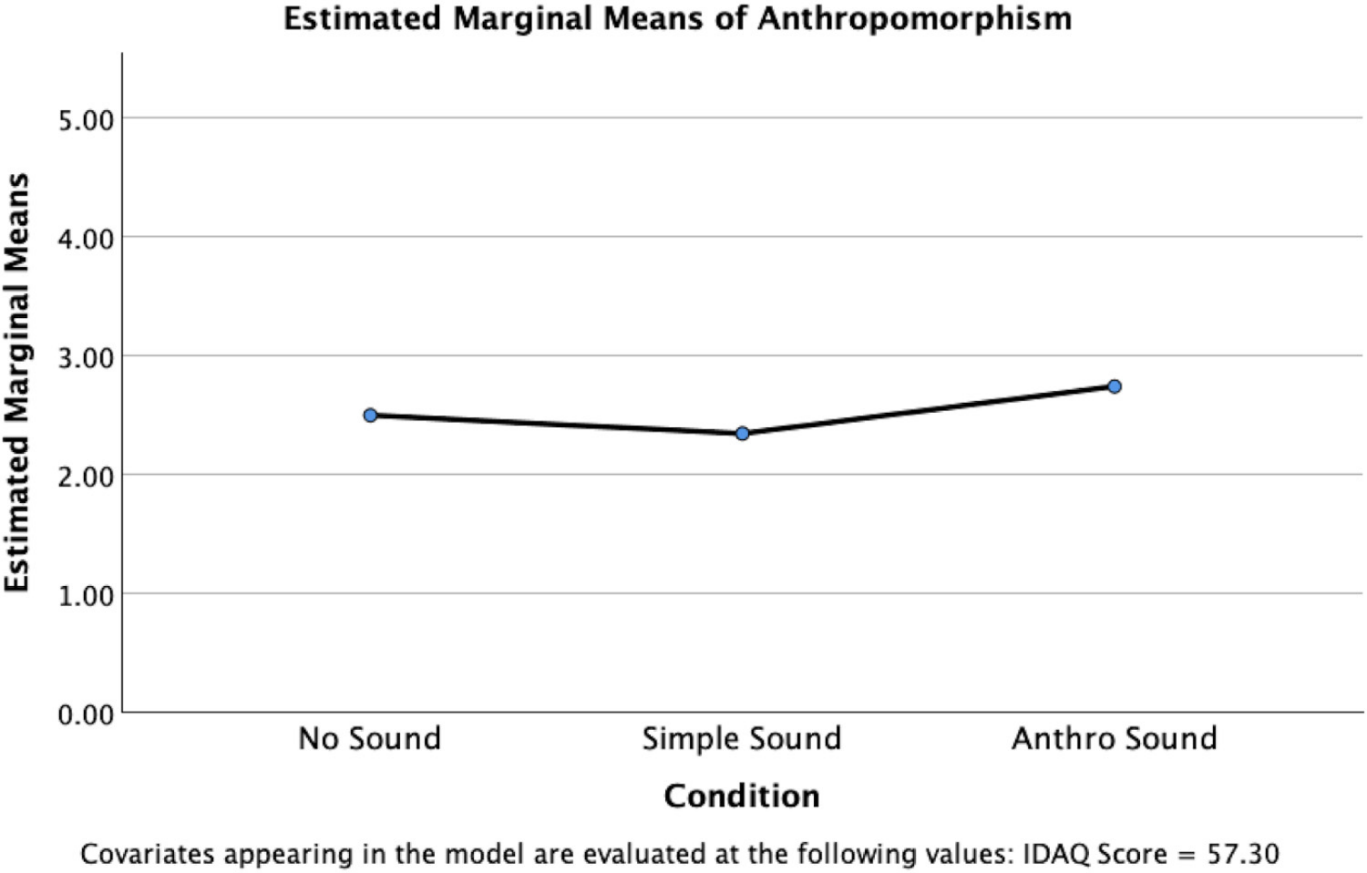
Estimated marginal means for anthropomorphism scores, controlling for IDAQ responses.

However, a significant difference between conditions was identified for anthropomorphism, *F*(2, 145) = 3.491, *p* = .033, partial *η*²=.046, indicating a small to medium effect size (Figure 5). Pairwise comparisons using Bonferroni adjustment revealed that the Anthro Sound condition (*M* = 2.742, SE = .108) had significantly higher scores than the Simple Sound condition (*M* = 2.345, SE = .111), *t*(145) = 2.629, *p* = .029. No significant differences were found between the No Sound condition (*M* = 2.499, SE = .111) and the Simple Sound condition, *p* = .954, or between the No Sound condition and the Anthro Sound condition, *p* = .359.

**Figure 6.**
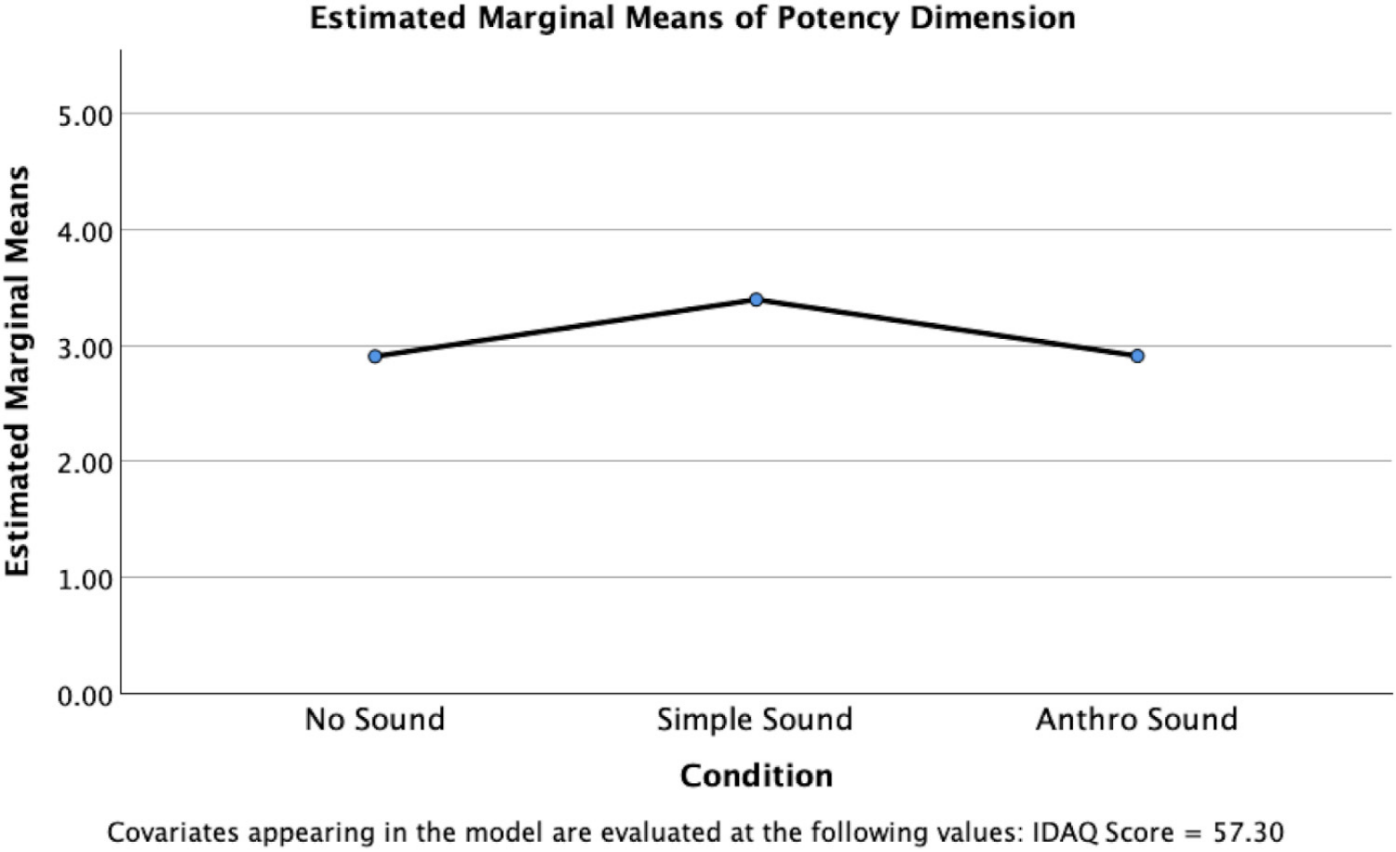
Estimated marginal means for potency dimension scores, controlling for IDAQ responses.

A significant difference between conditions was also identified for the potency dimension, *F*(2, 145) = 8.031, *p* ≤ .001, partial *η*²=.100, indicating a medium to large effect size (Figure 6). Pairwise comparisons using Bonferroni adjustment revealed that the Simple Sound condition (*M* = 3.398, SE = .099) had significantly higher scores than the Anthro Sound condition (*M* = 2.912, SE = .100), *t*(145) = 3.471, *p* = .002. The Simple Sound condition also had significantly higher scores than the No Sound condition (*M* = 2.908, SE = .103), *t*(145) = 3.434, *p* = .002. No significant differences were found between the Anthro Sound condition and the No Sound condition, *p* = 1.00.

### Discussion—Study One

For this study, hypothesis H1(a) (sound effects will contribute to an increase in both animacy and anthropomorphism), was partially met, but only in the case of one type of sound, and only for anthropomorphism. Hypothesis 1(b) (Human-based vocal sounds will be a greater contributor) offers us some insight into this result, showing that anthropomorphic sounds led to a slightly greater sense of anthropomorphism. However, the lack of results on animacy scores suggests that the presence of sound may be minimal overall to a sense of life-likeness of (typically inanimate) square shapes.

The only significant positive results in Study One were an increase in anthropomorphism scores for the Anthro sound, and an increase in potency score for the simple sound. The increase in perceived anthropomorphism for the Anthro sound is perhaps unsurprising, given the nature of the particular sound effects used: The other sound, a wood-block, has little connection to the sounds of many beings or creatures in the natural world, whereas the Anthro sound was more deliberately anthropomorphized, and closer to a human vocalization, since it was based on a human voice originally before being manipulated. People who had a stronger tendency to anthropomorphize were more likely to increase that tendency with sound, perhaps because they had less difficulty integrating the idea that a simple shape could be seen as a living creature. It is surprising, however, that animacy scores did *not* also increase in line with anthropomorphism. The fact that our anthropomorphism scores only increased for one type of sound suggests, however, that the *type* of sound, rather than the mere presence of sound, is a factor in our tendency to anthropomorphize.

The increase in potency score is also not surprising, given that the physical action that it would take to create a wood-block sound is one of smacking together two blocks, suggesting a strength inherent in the sound itself. Hearing a sound effect that is created through a powerful action as powerful (versus powerless), strong (versus weak), aggressive (versus submissive) and large (versus small) is expected. Due to the role of mirror neurons, our brains interpret the action behind a sound upon hearing it, and the action behind this sound was much stronger than no-sound or the Anthro sound (see, e.g., Collins 2011; Kim and Schachner 2021). Future studies could tease out further distinctions in responses to hearing different types of sounds. It would be interesting, for example, to explore if synthesized sounds mimicking the real-world sounds have the same kind of effect as actual recordings, and to explore if a participant can be trained to associate sounds with actions over time.

It is perhaps more important that sound had any impact, given the short length of this animation, and the single evocation of a sound. This suggests that sound effects, even when heard once, can have an impact on how we perceive a visual action, in line with existing multimodal research (e.g., Wan et al., 2020, who found high frequency sounds guided visual attention towards lighter coloured objects, or Sunaga, 2018, who found lower frequency sounds influenced perceived distances). Sekuler et al. (1997) found what is now referred to as the “auditory motion-bounce illusion,” whereby two identical visual targets will be perceived as crossing through each other in the absence of sound cues, but when a brief sound is added at the moment that the targets interact, a bias perception towards the targets bouncing off each other occurs. While in our study we did not explore such ambiguous crossing squares (rather, we created a visualized bounce), it would be interesting to see a replication where the bounce-promoting effect of sound at the point of coincidence is explored in terms of its impact on perceived anthropomorphism and animacy. Sekuler and Sekuler (1999) moreover express that the composition of the auditory stimulus (i.e., the type of sound effect) alters the effect considerably, aligning with our results here that show that perceptions of anthropomorphism are similarly impacted by the type of sound used.

The length of the animation (being just two squares bumping into each other once), and the single sound evoked, may not have been enough to make a significant difference to the anthropomorphism and animacy scores. Research has shown that prolonged exposure to multimodal stimuli can increase sensory integration (Stein et al., 2009), and extending the duration could allow for a deeper investigation into how participants’ perceptions evolve over time. This would be particularly valuable in settings where sound is not merely a one-off cue, but a continuous, evolving aspect of interaction, as in video games or virtual environments. As such, we increased the animation length and undertook a second study.

## Study Two: Heider-Simmel Recreation With Sound

The classic Heider and Simmel (1944) animation was recreated by the researchers using the same Javascript library as Study One, and then converted into a video so we could correctly synchronize and spatialize the sound. The original animation had no sound, and the influence of sound effects has not subsequently been explored until now. However, as described above, Marshall and Cohen (1988) explored the influence of a music soundtrack. They described that the process of attributing behavior to shapes may also be found in music listening, in that certain interactions between notes in music give rise to impressions such as warm, gentle, aggressive, etc. Certainly, there are many cross-modal phenomena that suggest that touch, color, shape, and sound may interact in our brains, whereby certain types of shapes can be associated with certain sounds, for example, sometimes known as the Bouba-Kiki effect, which also carries over into textures (see Fryer et al., 2014; Köhler, 1947). Moreover, melodic contours and dynamics in music such as accelerandos or crescendos may be interpreted alone as a form of movement (Leman, 2010). It is unclear, however, if non-musical sound effects would have the same impact on the perception of the animation. If our simple animation lasting just a few seconds couldn’t elicit a strong impact on anthropomorphism and animacy, would a longer animation, with different types of sounds, illustrate such an impact? We began with the same hypotheses as Study One, and added a hypothesis based on the binaural anthropomorphism work of Nielsen et al. (2015):

H1(a). Sound effects will contribute to an increase in both animacy and anthropomorphism, as evaluated by an increase in Godspeed scores.

H1(b). Human-based vocal sounds will be a greater contributor to this tendency than non-vocal sounds (real-world, or synthesized sounds).

H2. Binaurally spatializing sound will increase both animacy and anthropomorphism.

### Sounds

There were five sound conditions: no sound, Anthro sound, Simple sound, and then two binaural versions of the two sound conditions. We chose to add the binaural spatialization to see if binaural movement would have an impact, in the way that it did in the Nielsen et al. (2015) study described in the Introduction.

The sounds were again created by one of the researchers. The first sound, Anthro sound, can be described as anthropomorphized creature sounds. We included the addition of realistic door closing sounds and impact sounds. Secondly, we created Simple sounds, this time using non-realistic synthesized sounds using low resolution sound effects, roughly approximating a (1980s) Atari 2600, adapting synthesized “beeps” as a concept from Komatsu (2005). We then spatialized the two sound tracks using binaural sound. The first two, non-binaural tracks were in standard stereo with no panning (i.e., the sounds were in the same location in the stereo field).

The Anthro sounds condition consisted of two separate audio tracks. The sounds used in both tracks were created using similar methods to those described for the Anthro condition in Study One. For the first track, a “high voice” was created using the exact same steps, and was applied to all the voice recordings except for two, one of which was only increased in speed such that its pitch reached six semitones higher than the original, and the other was only increased until its pitch reached four semitones higher than the original. The rest were increased to eight semitones as described previously. The filtering and phaser effect remained constant. The speed of the recordings were preserved for this track using the “Preserve transients” warp mode within Ableton Live. On this track, an adjusted high-pass filter was applied, an amp simulator was added to apply some distortion and increase the upper harmonic content, as well as a phaser with different settings to the one applied on the “high voice” track. For the binaural condition with this Anthro sound set, the “high voice” roughly follows the path of the two smaller shapes, while the “low voice” follows the larger triangle shape.

The Simple sound condition used sounds created by the miniBit synthesizer developed by Audiothing (https://www.audiothing.net/instruments/minibit/). Some filtering and bitcrushing was applied using the built-in effects. There were also two tracks present in this condition: one following the same binaural path as the “high voice,” and the other following the path of the “low voice.” The track that followed the “high voice” of the smaller shapes also played higher pitched MIDI notes, either C2, B3, C4, or G4. The “low voice” track played exclusively D0. Additonally, slight automation adjustment was made to the volume of the “high voice” track at one point during its runtime.

Foley (realistic recorded) sound effects created by the research team also accompanied all sound conditions. This included door handle and creak sound effects as well as impacts between the shapes, impacts between the shapes and the wall, the walls being broken, and the pieces of the walls being pushed around. They were also placed in, or moved around in binaural space, for the appropriate conditions.

Binaural placement was done using the dearVR MICRO plugin (https://www.dear-reality.com/products/dearvr-micro). The elevation control was used to represent the vertical position of whichever element of the animation was being followed by the sound, while the azimuth control represented the horizontal position. The azimuth was also limited to between −90° and 90° because the animation was in two dimensions, meaning that sounds should never be perceptually behind the listener. Likewise, the elevation control was limited to between −50° and 50°. All instances used reflections model of the “drapes” room preset, with the level at 0 db and the size at 10.0 m. The focus was set to 100 and the width was set to 5%. The head-related transfer function (HRTF) used was the default “dearVR” function. Finally, some limiting was applied and the bass frequencies below 90.5 Hz were summed to mono for all tracks across all conditions.

In the non-binaural sound conditions, the sounds were evenly split in the stereo field, and there were no panning effects used, resulting in these sounds remaining essentially static. There was still some (albeit limited) use of the stereo field to position the sounds, and there are some sounds that clearly move between the two channels. These sounds were normalized to the same volume level (−3 dB).

### Questionnaires

Study Two was constructed in the same way as Study One with regards to the questionnaires and structure of the surveys. However, when completing the GSQ, participants were specifically asked about their perception of the *large triangle* in the animation.

### Participants

Participants were required to speak English, and be a minimum age of 18. We recruited 250 participants for this study, after removing three for failing attention checks. Participants took approximately eight minutes to complete the study and were paid £1.50 (GBP). A total of 47 participants were in the No Sound condition, 46 in the Anthro Sound condition, 44 in the Simple Sound condition, 58 in the Anthro Sound Binaural condition, and 55 in the Simple Sound Binaural condition.

In terms of audio device used to complete the study, 30 participants used external speakers, 22 used built-in desktop speakers, 74 used built-in laptop speakers, 11 used mobile phone speakers, and 113 used headphones. A total of 237 participants reported no known hearing loss, 8 reported hearing loss, and 6 were unsure. These factors had no statistically significant impact on any of their responses, so the statistical analysis included all participants.

There were 59 participants in the 18–24 age group, 122 in the 25–34 age group, 44 in the 35–44 age group, 18 in the 45–54 age group, and 7 in the 55 + age group. An ANOVA showed no significant differences in IDAQ scores between different age groups (*F*(4, 245) = .506, *p* = .731, *η*^2^=.008). 28 participants were from North America, 217 participants were from Europe, and 5 participants were from other regions. Likewise, an ANOVA showed no significant differences in IDAQ scores between regions (*F*(2, 247) = .510, *p* = .601, *η*^2^=.004).

There were 113 participants who identified as women, 133 identified as men, and 4 identified as other. A one-way ANOVA revealed a *p*-value of .054 between groups, close to the threshold for significance (*F*(2, 247) = 2.957, *p* = .054, *η*^2^=.023). We therefore ran a Tukey's HSD, which revealed a significant difference between men (*M* = 44.09, SD = 20.658) and women (*M* = 50.74, SD = 22.339), *t*(247) = 2.431, *p* = 0.042, although no other significant differences were found between the groups. In other words, women were slightly more likely to anthropomorphize according to their IDAQ scores. Women's mean scores were also higher, on average, than participants who identified as other (*M* = 48.67, SD = 16.215), although this difference was insignificant.

### Results of Study Two

**Figure 7.**
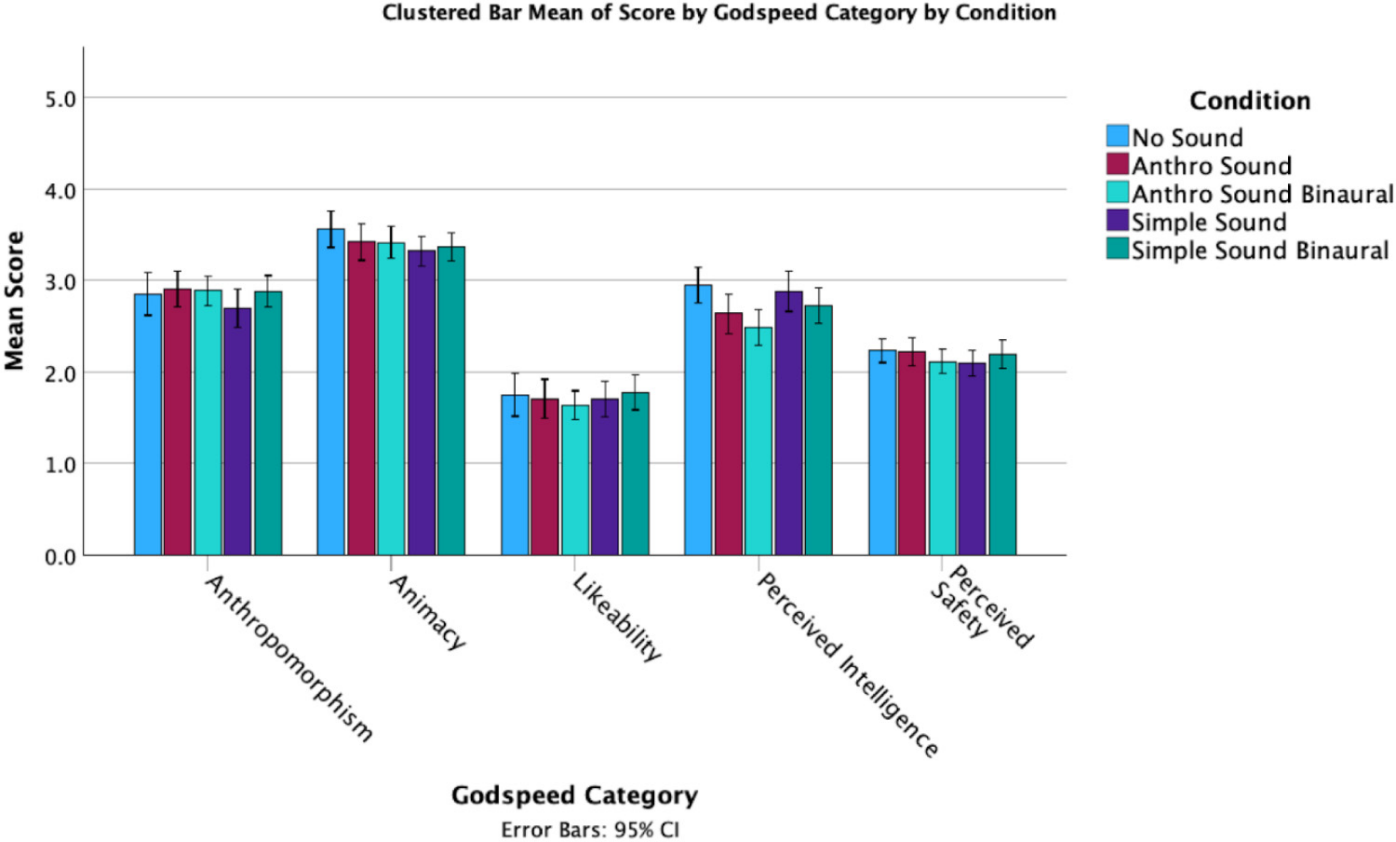
Overall means of godspeed scores by condition.

**Figure 8.**
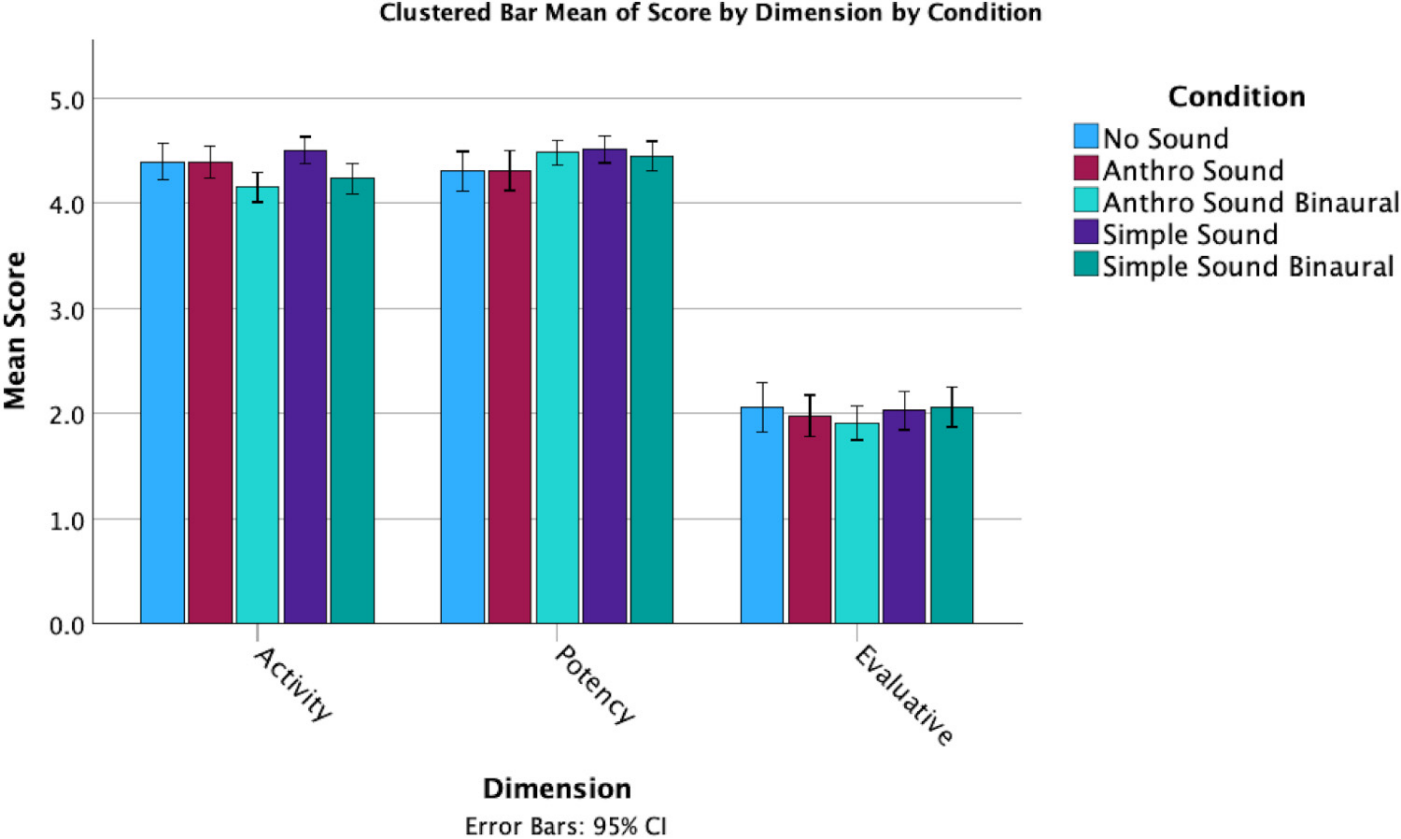
Overall dimension means for each condition.

Exploring the overall means, it appears as if the No Sound condition has a higher animacy score than any of the sound conditions (the No Sound condition had the highest mean animacy score (*M* = 21.38, SD = 4.126), while the Simple Sound condition had the lowest mean animacy score (*M* = 19.93, SD = 3.136)), although No Sound scores were lowest on potency (Figures 7 and 8).

The statistical analysis was again carried out in SPSS (IBM Corp, 2023). Scatterplots along with Pearson Correlation coefficients indicated that there were no significant linear relationships present between the covariate, IDAQ scores, and the dependent variables: anthropomorphism, *r*(248) = −.061, *p* = .337, animacy (*r*(248) = −0.37, *p* = .555), likeability (*r*(248) = −.039, *p* = .538), perceived intelligence (*r*(248) = −.055, *p* = .538), perceived safety (*r*(248) = −.049, *p* = .383), evaluative dimension (*r*(248) = −.048, *p* = .450), potency dimension (*r*(248) = −.075, *p* = .450), and activity dimension (*r*(248) = −.081, *p* = .203). Due to this violation of the assumption of linearity, we determined that a one-way ANOVA test was more appropriate than an ANCOVA for this analysis, leading to a different approach to this statistical analysis than in Study One.

The normality of residuals was assessed using both Shapiro-Wilk tests and visual inspection of normal Q–Q plots. For several variables, the Shapiro-Wilk test indicated an approximate normal distribution of residuals: anthropomorphism (*W* = .991, *p* = .130), animacy (*W* = .993, *p* = .244), and perceived intelligence (*W* = .990, *p* = .068). However, for perceived safety (*W* = .983, *p* = .005) and the evaluative dimension (*W* = .973, *p* < .001), the Shapiro-Wilk test suggested a violation of normality. Despite this, visual inspection of Q–Q plots indicated that the deviations were minimal and did not follow clear patterns of skewness or kurtosis, suggesting approximate normality. Following the considerations outlined in Study One regarding the sensitivity of the Shapiro-Wilk test and robustness of ANOVA tests (Blanca Mena et al., 2017) (see section “Results of Study One”), an ANOVA was still conducted for variables that appeared approximately normal upon inspection of the Q−Q plot.

For the remaining variables, significant deviations from normality of the residuals were confirmed by both Shapiro-Wilk tests and visual inspections: likeability (*W* = .909, *p* ≤ .001), potency dimension (*W* = 887, *p* ≤ .001), and activity dimension (*W* = .939, *p* < .001). Accordingly, the non-parametric Kruskal-Wallis H test was used to analyze these variables.

Levene's test confirmed homogeneity of variances for all variables analyzed using ANOVA: anthropomorphism (*F*(4, 245) = 1.882, *p* = .114), animacy (*F*(4, 245) = 1.593, *p* = .177), perceived intelligence (*F*(4, 245) = .459, *p* = .766), perceived safety (*F*(4, 245) = 1.308, *p* = .268), and evaluative dimension (*F*(4, 245) = 1.674, *p* = .156).

#### ANOVA Results

One-way ANOVAs revealed no statistically significant differences between conditions for anthropomorphism (*F*(4, 245) = .753, *p* = .557, *η*²=.012), animacy (*F*(4, 245) = .967, *p* = .426, *η*²=.016), perceived safety (*F*(4, 245) = .758, *p* = .553, *η*²=.012), or evaluative dimension (*F*(4, 245) = .495, *p* = .740, *η*²=.008).

**Figure 9.**
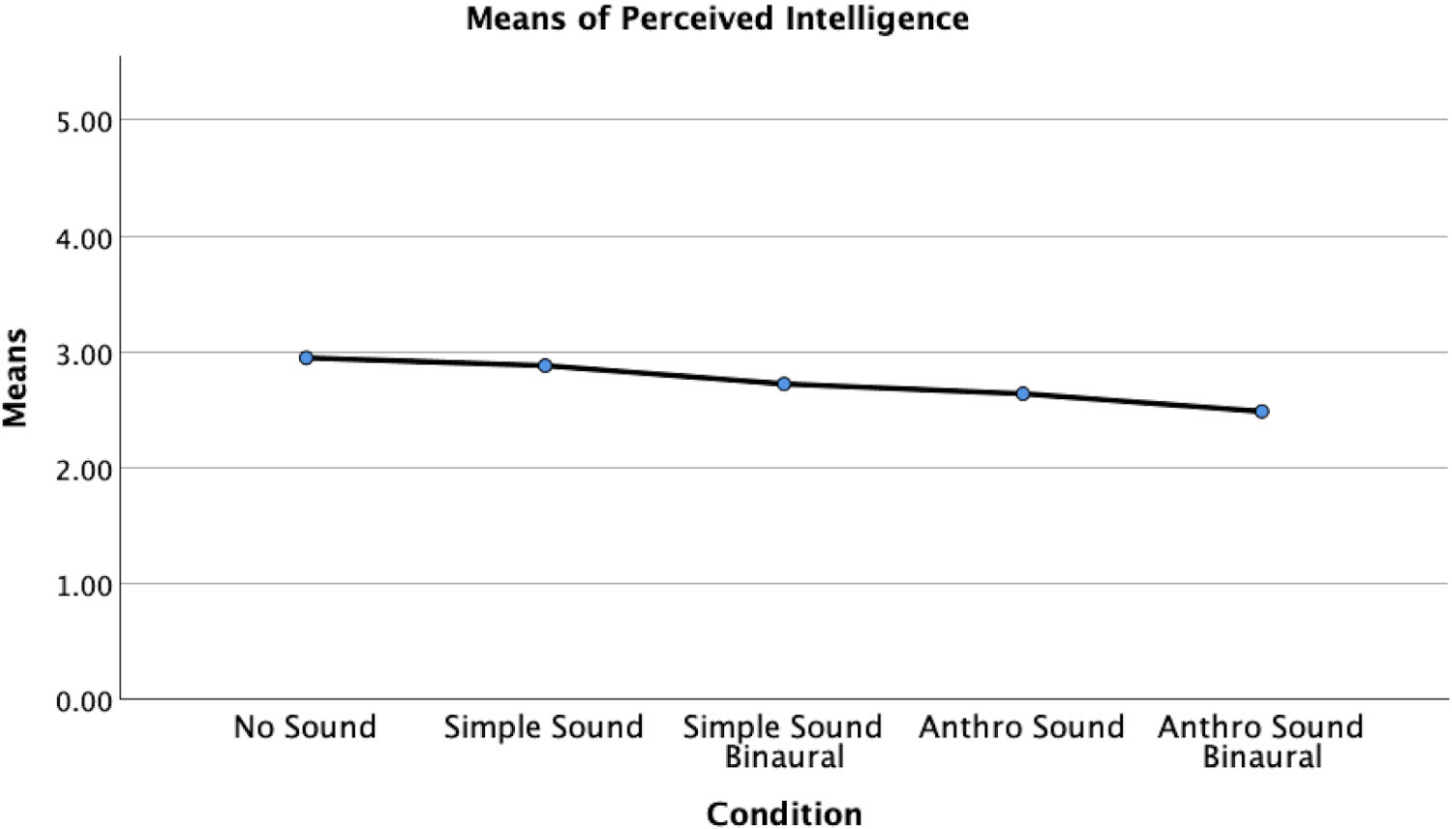
Means of perceived intelligence by sound condition.

However, a significant difference between conditions was identified for perceived intelligence, *F*(4, 245) = 3.433, *p* = .009, *η*^2^ = .053, indicating a small to medium effect size (Figure 9). Post hoc tests using Bonferroni adjustment revealed that the No Sound condition (*M* = 2.949, SD = .665) had significantly higher perceived intelligence scores than the Anthro Sound Binaural condition (*M* = 2.486, SD = .742), *t*(245) = 3.282, *p* = .012.

No significant differences were found between the No Sound condition and Anthro Sound condition (*M* = 2.639, SD = .732), *p* = .386; the No Sound condition and Simple Sound condition (*M* = 2.881, SD = .723), *p* = 1.00; or the No Sound condition and Simple Sound Binaural condition (M = 2.734, SD = .721), *p* = 1.00. Additionally, no significant differences were found between the Anthro Sound condition and the Anthro Sound Binaural condition, *p* = 1.00; the Simple Sound condition and Anthro Sound condition, *p* = 1.00; the Simple Sound condition and Anthro Sound Binaural condition, *p* = .063; the Simple Sound condition and Simple Sound Binaural condition, *p* = 1.00; the Simple Sound Binaural condition and Anthro Sound condition, *p* = 1.00; or the Simple Sound Binaural condition and Anthro Sound Binaural condition, *p* = .803.

#### Kruskal-Wallis Results

As a reminder, the Kruskal-Wallis test was used for likeability, potency, and activity dimension scores due to significant deviations from normality. Results from the test indicated no statistically significant differences between conditions for likeability (*H*(4) = .722, *p* = .949, *ε*²=.00) or the potency dimension (*H*(4) = 3.361, *p* = .499, *ε*² = .00).

**Figure 10.**
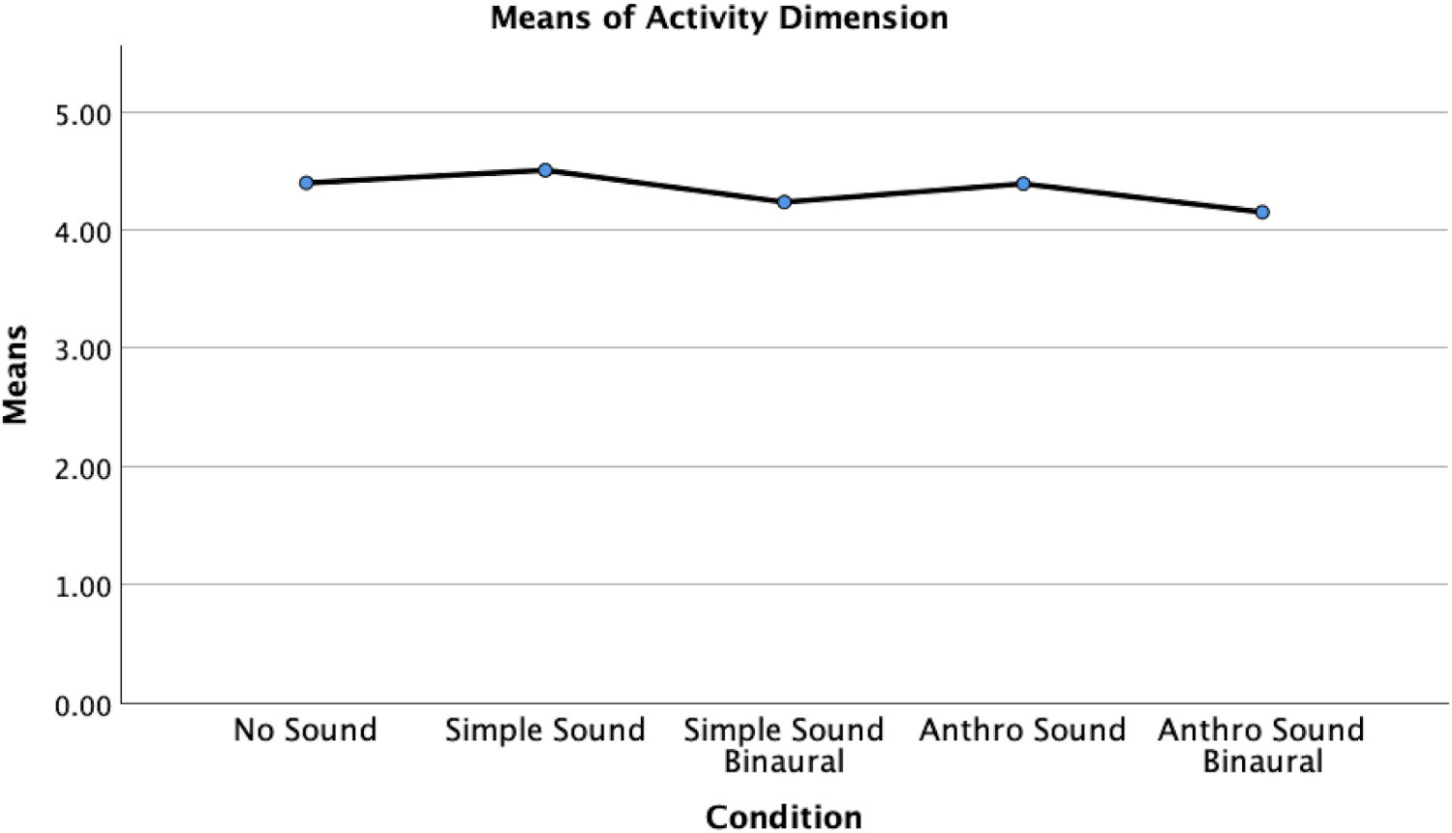
Means of the activity dimension, by sound condition.

However, a significant difference between conditions was identified for the activity dimension, *H*(4) = 16.708, *p* = .002, *ε*²=.052, indicating a small to medium effect size (Figure 10). Post hoc analyses using Mann-Whitney *U* tests were conducted to examine pairwise differences between the conditions. The Bonferroni-adjusted significance level was set at *α*=0.05/10 = 0.005. Results indicated that the activity dimension scores in the No Sound condition (Mdn = 4.50) were significantly higher than scores in the Anthro Sound Binaural condition (Mdn = 4.125), *U* = 923.50, *z* = −2.865, adjusted *p* = .04, r = .28, indicating a medium effect size. Additionally, scores in the Simple Sound condition (Mdn = 4.50) were significantly higher than scores in the Anthro Sound Binaural condition, *U* = 778.00, *z* = −3.409, adjusted *p* ≤ .01, *r* = .340, indicating a medium to large effect size.

No significant differences in activity dimension scores were found between the No Sound condition and the Anthro Sound condition (Mdn = 4.50), *U* = 1022.00, *z* = −.461, adjusted *p* = 1.00, *r* = .049; the No Sound condition and the Simple Sound condition, *U* = 970.500, *z* = −.513, adjusted *p* = 1.00, *r* = .054; the No Sound condition and the Simple Sound Binaural condition (Mdn = 4.25), *U* = 1002.00, *z* = −1.976, adjusted *p* = .480, *r* = .196; the Anthro Sound condition and the Anthro Sound Binaural condition, *U* = 950.500, *z* = −2.540, adjusted *p* = .110, *r* = .249; the Anthro Sound condition and the Simple Sound condition, *U* = 905.00, *z* = −.880, adjusted *p* = 1.00, *r* = .093; the Anthro Sound condition and the Simple Sound Binaural condition, *U* = 1040.50, *z* = −1.552, adjusted *p* = 1.00, *r* = .154; the Anthro Sound Binaural condition and the Simple Sound Binaural condition, *U* = 1433.50, *z* = −.938, *p* = 1.00, *r* = .088; or the Simple Sound condition and the Simple Sound Binaural condition, *U* = 869.50, *z* = −2.431, adjusted *p* = .15, *r* = .244.

Overall, the study found that different sound conditions had a significant impact only on the perceived intelligence and activity dimensions of the Godspeed questionnaire, and not, as we predicted, on anthropomorphism and animacy. The IDAQ score did not significantly influence the outcomes in most dimensions, except for a slight effect observed in the activity dimension.

### Discussion—Study Two

The results of Study Two were again unexpected. Sound had no significant impact on animacy and anthropomorphism, failing to confirm Hypothesis 1(a). Moreover, the type of sound did not impact this tendency in this study, failing to confirm Hypothesis 1(b). Surprisingly, sound had no significant impact on most of the factors we explored, with only a minor impact on perceived intelligence (lowering the perceived intelligence, versus no sound), and having a small and inconsistent impact on the activity dimension.

It was interesting that our attempts to create an anthropomorphic sound resulted in the lowest perceived intelligence scores. This could mean that the incongruence in attributing anthropomorphic sounds to inanimate shapes leads to negative perceptions. If for instance our participants associated such sounds with particular characters in films, or simply associated those sounds with living creatures, the incongruence of hearing the sounds with simple shapes may have led to a greater increased negative response than no sound at all.

In the two areas where there were statistically significant differences (perceived intelligence and activity dimension), the scores were lower in the binaural versions than in standard stereo, failing to confirm Hypothesis 2 (“Binaurally spatializing sound would increase both animacy and anthropomorphism”). We suggest that it is possible that the lack of experience with binaural sound (there are almost no films that use binaural sound) led to lower response due to the unfamiliarity of the effect in animation. We believe we did see enough minor differences between the binauralized and stereo versions of the files to warrant further exploration in this area. In particular, it may have been useful to have a continuous sound that tracks the movement of the shapes that moves around in the stereo field with the object, rather than the on-off sound effects we used. Our approach to binauralization may have also played a role in the lack of significant differences with binaural sound, and an animation where the visual movement is more exaggerated, or the distance between shapes greater, leading to greater and more emphasis on the separation between channels may be worth exploring in further studies. We also took our participants at their word that they were using headphones (and that these were indeed stereo headphones), and in future, the use of headphone checks that test to ensure correct stereo separation could be used, although this seems less likely a factor (e.g., Woods et al., 2017).

The lack of impact that sound had on anthropomorphism, animacy, or indeed most of the dimensions tested was not consistent with Marshall and Cohen's study that used music, rather than sound effects (1988). Their study found that the Activity and Potency dimensions of the larger triangle increased without music, while the Evaluative dimension decreased without music. Our results showed perceived Activity increased with stereo sound, but decreased with binaural sound. However, Marshall and Cohen did find that the *type* of music also impacted the results. This suggests that, in line with our findings that the type of sound can impact results, it is not the mere presence of auditory stimuli, but the type of auditory stimuli that is perhaps most important to our interpretation of visual stimuli. This result is consistent with other research that has shown different types of sound influence gaze (Song et al., 2013), and influences situational awareness responses to interface sounds in games (Robb et al., 2017).

## General Discussion

We presented here two studies that have explored the impact of sound on perceptions of animacy and anthropomorphism in simple animated shapes. In both studies, several insights emerged, though the results were somewhat mixed, highlighting both the potential and the limitations of sound in influencing these perceptions. Our results were, for the most part, contrary to our expectations and hypotheses. It appears, based on our studies, that the sound effects we chose had a limited impact on perceptions of animacy and anthropomorphism when it comes to simple shapes. Across both studies, the findings suggest that it is not merely the presence of sound, but the specific characteristics of sound that affect how we perceive visual stimuli. While some sounds influenced certain dimensions, the results were not consistent across sound conditions, pointing to a complex interaction between auditory and visual cues.

There are, however, many possible explanations for our lack of definitive results. Firstly, the movements of the shapes, especially in Study Two, was already shown in the existing literature to elicit anthropomorphic sentiments. Perhaps sound simply does not add more to these perceptions. Another explanation for these findings is the specific nature of the sounds used in this study. It is possible that the sounds were not sufficiently engaging or relevant to elicit strong responses from participants. There may have been associations our listeners had with the sounds that we are not aware of, and adding qualitative data to such studies may be useful in the future. For instance, if the participants heard our anthropomorphic sounds as being similar to existing characters in film, they may have had difficulty associating them with the shapes in our experiment, and the incongruence may have led in part to the lack of meaningful results.

In Study One, the findings indicated that specific types of sound, particularly anthropomorphized (human-like) sounds, could enhance the perception of anthropomorphism, but not animacy. This suggests that while sound can contribute to how we attribute human-like qualities to inanimate objects, the type of sound is crucial, and a single sound effect alone may not be sufficient to increase the sense of animacy. The impact of the Anthro sound on anthropomorphism, but not on animacy, suggests that the anthropomorphized nature of the sound played a critical role. Future studies could explore the degree of anthropomorphization in sounds selected on their own prior to implementation. Research has shown that even minimal anthropomorphism in design can shift how people perceive and interact with technology (Waytz et al., 2010), so testing the boundary conditions of how much sonic anthropomorphism is needed to influence perception could be highly informative. Additionally, it would be interesting to explore the role of emotional tone in the anthropomorphized sounds, as emotional prosody in speech has been shown to affect perception and interaction with artificial agents (Pell & Skorup, 2008).

The results did suggest that the type of sound selected did play a role in perception, rather than the mere presence of sound tied to the visual action. The increase in potency scores tied to the physical characteristics of sounds (such as the wood-block sound) demonstrates how certain sound qualities are interpreted in terms of strength and power, underscoring the role of sound in influencing perceptions of physical attributes. Further work should delve into many other different types of sound and the impact that different types of sound effects may have on a variety of perceptual aspects.

In Study Two, we expected to see stronger results due to the extended length of the animation and the introduction of binaural sound. The lack of significance in the spatialized sound was also surprising, and it could be that there was not enough movement of sounds to make a significant difference to the results. If the shapes had made a constant sound that panned with their movement, for instance, we may have witnessed different results, and future research could explore that aspect further. Moreover, we did not test for headphone use, and instead relied on their self-reported use. There are headphone checks that would have added considerable time to the survey, but could be used in future studies to ensure they have adequate stereo separation (e.g., Milne et al., 2020; Woods et al., 2017). However, sound had no significant impact on animacy or anthropomorphism in this context, and only minor effects on perceived intelligence and perceptions of activity. Additionally, testing how sound movement influences gaze direction and attention (Song et al., 2013) could clarify the role of sound in modulating focus on different visual elements. Interestingly, binaurally spatialized sound produced lower perceived intelligence and activity scores, raising questions about the role of binaural audio in enhancing perception, which warrants further investigation. It would be interesting, for example, to test the sound alone in binaural and standard stereo versions and see if listeners noticed a difference in our files, replicating the Nielsen et al. (2015) study.

The interaction between sound and image is complex, and incongruence between the visual image and auditory information can alter perception. Sound is often integrated with visual image to create an overall perception, but visual stimuli typically take precedence, which is known as the visual dominance effect, or Colavita effect (see, e.g., Spence 2009). Our sounds might not have been impactful enough to override the visual stimuli. The simplicity of the animation may have also played a role, and future studies could explore more complex visual animations to see if sound impacts animacy or anthropomorphism in more anthropomorphic visuals.

The results may indicate the importance of congruence between sound and visual stimuli when it comes to anthropomorphism and animacy. Future studies should consider the nuances of sound-visual congruence to fully understand the effects of sound on this perception. It would be interesting to repeat the study with actual characters rather than simple shapes to explore the impact that sound has. In the real world, triangles and squares of course make no sound: would other more complex creature-shaped shapes, and/or visual characters, have a different result when combined with sound? Would repeating the study with three dimensional shapes and continuous sound, including background sound, have a greater influence on the results? Previous work has shown that cross-modal congruence between auditory and visual stimuli plays a crucial role in perception. Studies on cross-modal correspondences between sound frequency and object properties (Gallace et al., 2012) provide strong support for the idea that congruence between auditory and visual cues enhances cognitive processing and perception. It could be that an incongruence between 2D shapes and highly spatialized 3D audio impacted our results. Future research could also investigate how the congruence between auditory characteristics (e.g., pitch, tempo, timbre) and visual features (e.g., shape, color, motion) influences perceptions of animacy and anthropomorphism. Specifically, testing whether more congruent auditory-visual pairs lead to higher levels of perceived animacy and anthropomorphism could help clarify how our brains integrate multimodal stimuli.

Running these studies online means we did not control for other potential external variables. In music perception testing, studies have shown the validity of remote testing (Honing & Ladinig, 2008; Peng et al., 2022). When it comes to auditory psychophysics, however, online/remote testing has been mostly avoided (Eerola et al., 2021), since there are a considerable number of factors beyond the experimenter's control. Users may not understand the impact of background noise on the study, and be subject to background noise interference. It is difficult to know the hearing capabilities of end users, the volume of their playback devices, or the quality of their equipment when using remote testing (Peng et al., 2022; Zhao et al., 2022). We did not control for volume levels or external distractions and noise, although the differences in technology used did not appear to make any difference. Noise and volume level may have had some impact if sound levels were low for some participants (Wycisk et al., 2023; Yang and Kang, 2005; Zhao et al., 2022). It is not possible to easily calibrate to consistent volume levels for participants in remote studies, however studies completed in person could mitigate this factor by having a strict volume setting. Some attempts at calibration may be necessary for these types of tests, and collecting information about the specific make and model of equipment used by participants may also be beneficial to future studies.
