# Erratum to “Sound effects have only minor contribution to perceptions of anthropomorphism and animacy of simple animated shapes”

**DOI:** 10.1177/20416695251356615

**Published:** 2025-07-10

Collins K. C., Murad M., Manji A. (2025). Sound effects have only minor contribution to perceptions of anthropomorphism and animacy of simple animated shapes. I-Perception, 16. https://doi.org/10.1177/20416695251315382

In this article, the first author's name was originally listed incorrectly as "K Collins". It has now been corrected to "K C Collins”.

